# Development of Nanocrystal Compressed Minitablets for Chronotherapeutic Drug Delivery

**DOI:** 10.3390/ph15030311

**Published:** 2022-03-04

**Authors:** Nagaraja Sreeharsha, Nimbagal Raghavendra Naveen, Posina Anitha, Prakash S. Goudanavar, Sundarapandian Ramkanth, Santosh Fattepur, Mallikarjun Telsang, Mohammed Habeebuddin, Md. Khalid Anwer

**Affiliations:** 1 Department of Pharmaceutical Sciences, College of Clinical Pharmacy, King Faisal University, Al-Hofuf, Al-Ahsa 31982, Saudi Arabia; 2 Department of Pharmaceutics, Vidya Siri College of Pharmacy, Off Sarjapura Road, Bangalore 560035, Karnataka, India; 3 Department of Pharmaceutics, Sri Adichunchanagiri College of Pharmacy, Adichunchanagiri University, B.G. Nagar 571448, Karnataka, India; pgoudanavar01@gmail.com; 4 Department of Pharmaceutics, Annamacharya College of Pharmacy, New Boyanapalli, Rajampet 516126, Andhra Pradesh, India; posina.anitha26@gmail.com; 5 Department of Pharmaceutics, Karpagam College of Pharmacy, Coimbatore 641032, Tamilnadu, India; ramkanth@kcp.edu.in; 6 School of Pharmacy, Management and Science University, Seksyen 13, Shah Alam 40100, Selangor, Malaysia; 7 Department of Medicine, College of Medicine, King Faisal University, Al-Ahsa 31982, Saudi Arabia; mvtelsang@kfu.edu.sa; 8 Department of Biomedical Sciences, College of Medicine, King Faisal University, Al-Ahsa 31982, Saudi Arabia; hmohammed@kfu.edu.sa; 9 Department of Pharmaceutics, College of Pharmacy, Prince Sattam Bin Abdulaziz University, Al-Alkharj 11942, Saudi Arabia; m.anwer@psau.edu.sa

**Keywords:** chronotherapeutic system, nanocrystals, design of experiments, Box–Behnken, valsartan

## Abstract

The present work aimed to develop a chronotherapeutic system of valsartan (VS) using nanocrystal formulation to improve dissolution. VS nanocrystals (VS-NC) were fabricated using modified anti-solvent precipitation by employing a Box–Behnken design to optimize various process variables. Based on the desirability approach, a formulation containing 2.5% poloxamer, a freezing temperature of −25 °C, and 24 h of freeze-drying time can fulfill the optimized formulation’s requirements to result in a particle size of 219.68 nm, 0.201 polydispersity index, and zeta potential of −38.26 mV. Optimized VS-NC formulation was compressed (VNM) and coated subsequently with ethyl cellulose and HPMC E 5. At the same time, fast dissolving tablets of VS were designed, and the best formulation was loaded with VNM into a capsule size 1 (average fill weight—400–500 mg, lock length—19.30 mm, external diameter: Cap—6.91 mm; Body—6.63 mm). The final tab in cap (tablet-in-capsule) system was studied for in vitro dissolution profile to confirm the chronotherapeutic release of VS. As required, a bi-pulse release of VS was identified with a lag time of 5 h. The accelerated stability studies confirmed no significant changes in the dissolution profiles of the tab in cap system (*f*_2_ similarity profile: >90). To conclude, the tab in cap system was successfully developed to induce a dual pulsatile release, which will ensure bedtime dosing with release after a lag-time to match with early morning circadian spikes.

## 1. Introduction

Untimely release of the un-quantified drug may lead to numerous deaths and hospitalization, as evidenced by miscellany studies across the world [1]. Circadian rhythms can directly impact the usual body functioning, and they are also accountable for the kinetics of the administered drug product [2]. Many physiological functions and hormones abide by the circadian cycles [3]. Basic understanding and profound knowledge of chronobiology may aid in developing novel technologies for maximizing drug delivery. Recently, much attention has been drawn to the advancement of chronotherapeutic drug delivery systems. Following chronobiology, these systems can efficiently release the desired amount of drug at the precise time and exhibit action at the target site [4]. The release pattern of the drug through this system is noted as sigmoid concerning time with a specific time gap to correlate with the associated disease. Chronobiology exerts influence on chronic conditions such as angina [5,6,7], asthma [8,9,10], hypertension, arthritis [11,12], etc., and the circumstances of such diseases get exacerbated in the medial night or prime hours in the morning [13,14,15].

Some cardiovascular incidents can often appear in days and manifest diurnal habits [16,17]. A high rush of blood pressure at early hours in the morning was noted to have an association with the development of hemorrhagic stroke and sudden cardiac deaths [18,19]. The increased activity of plasma renin and noradrenaline hormones in the morning hours can cause vasoconstriction in the coronary artery, thus leading to greater risk [20].

Valsartan (VS) is a long-acting angiotensin blocker that was reported to possess antihypertensive properties to control ambulatory blood pressure without disturbing its diurnal nature in the patients. Plasma norepinephrine levels and plasma renin activity are elevated in the morning. Both hormones can induce coronary vasoconstriction [21,22]. Thus, the renin-angiotensin-aldosterone system is activated in the morning and may contribute to morning blood pressure surges and morning increases in cardiovascular risk. The pharmacokinetics of valsartan showed that valsartan peak plasma concentration is reached in 2 to 4 h after conventional drug dosage form administration. VS shows an average elimination half-life of about six h. VS’s traditional drug delivery system is inappropriate for drug delivery, as they cannot be administered just before the symptoms are worsened. Bedtime dosing of conventional drug dosage of VS will not provide a sufficient therapeutic plasma drug concentration at the early hours of the morning. VS has a BP-lowering effect for controlling ambulatory blood pressure levels without disrupting its diurnal variation in hypertensive patients.

The differences in chronotherapeutic effects of angiotensin-II receptor blockers, valsartan, and olmesartan in hypertensive patients with non-dipper blood pressure patterns during valsartan in the morning were studied by Kentaro Ushijima et al., and Pena-Seijo et al. [23,24]. Valsartan pulsatile capsule dosage form for controlled delivery was initially formulated by Nayak et al. [25]. Another study of chronomodulated, time-clock pulsatile tablets of valsartan to release the drug after a specific lag time, independent of the gastrointestinal pH in its absorption window to cope with the human body’s circadian rhythm, was designed successfully [26]. However, all of these studies are not focused on the solubility enhancement or bi-pulse release of the chosen drug. Nevertheless, no attempt was made to develop a system for angiotensin II receptor blockers that can depot drugs at a defined time and exhibit optimum effect at prime hours in the morning [25].

The poor solubility and the variation between fed and fasted state of VS limit the formulation approaches and patient compliance. To enhance patient compliance and bioavailability of the drug, many studies have been conducted with nanotechnological processes, such as lipid-based nano delivery systems, self-emulsifying drug delivery systems, and nanocrystal containing solid pharmaceutical dosage forms [27,28]. Nanotechnology in pharmaceutical development is essential to enhance the oral bioavailability of the drug in schizophrenia treatment. Nanoparticular systems that comprise particulates between 10- and 1000-nm sizes increase saturated solubility and stability of low water-soluble drugs as well as oral bioavailability differences between fasted- and fed-state conditions [29]. Nanosuspensions or nanocrystals among nanoparticulate systems have become more popular concerning the suitability for scale-up procedures, the minimum amount of excipients, and enhancement of apparent solubility due to their small particle size [30]. To improve the quality of the formulations and reduce the number of experiments during the formulation development, the design of experiment approach can be used effectively [31,32]. Response surface methodology (RSM) is to be performed after precisely selecting variables that significantly affect the responses. It is usually achieved with screening designs such as factorial designs. The first-order designs assess the variable’s linear functions on the output. Still, the design could not estimate the curvature. However, second-order designs such as the Box–Behnken design (BBD) and central composite design, can evaluate the variables’ curvature interaction and present it in terms of a quadratic equation. Among them, BBD is preferred since it needs minimum experimental trials and does not include any points at the edges of the cubic region. Initially, a model was generated with a traditional RSM design [33,34]. The model was fitted after performing the experimental runs by incorporating the responses. The RSM statistical design based on the BBD design is advantageous over the typical method to optimize the several critical factors for the proposed study to estimate their influences [35,36,37].

Therefore, we made an effort to develop a capsule-based pulsatile drug delivery system of VS for pulsatile delivery. Fabrication of the pulsed-release capsule device comprises a fast-dissolving system and compressed nanocrystals formulation coated with ethyl cellulose and hydroxy methylcellulose (HPMC) E5. The coated body of the capsule is attached to a water dissolvable or uncoated cap. The effect of various factors corresponding to the concentration of poloxamer 188, freezing temperature, and freeze-drying time on nanocrystals preparation was investigated.

## 2. Results and Discussion

### 2.1. Compatibility Studies

**Figure 1:**
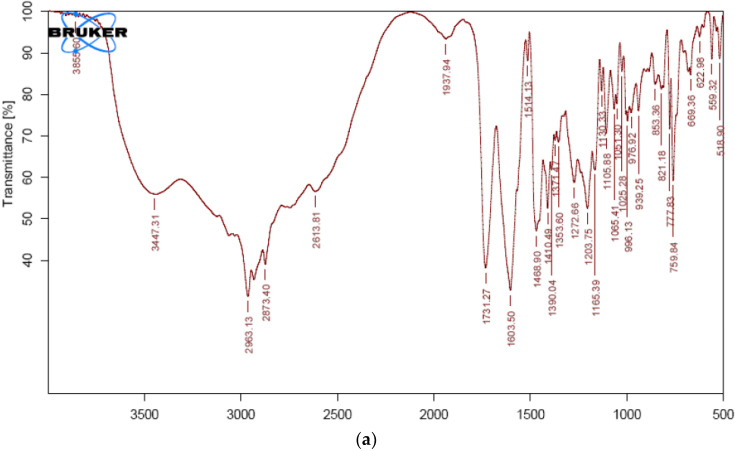

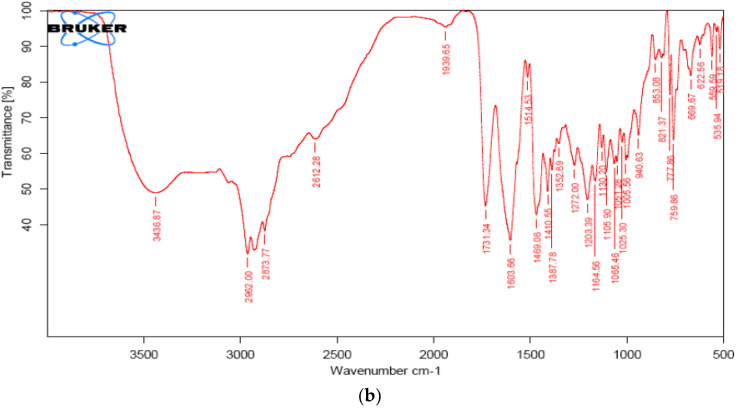
FTIR Spectrum of (**a**) pure VS and (**b**) physical mixture of VS formulation.

FTIR analyses of VS and physical mixture of VS with all of the formulation ingredients were carried out, and the spectra were shown in Figure 1a,b. VS showed characteristic peaks at 3447 cm^−1^ due to N-H stretch; 2963 cm^−1^ corresponding to C-H stretch; 1603 cm^−1^ representing amide group etc. FTIR spectrum of VS and formulation confirms all of the characteristic peaks of VS without any distinct alterations (Table 1). Thus, demonstrating the compatibility of VS with selected excipients.

**Figure 2:**
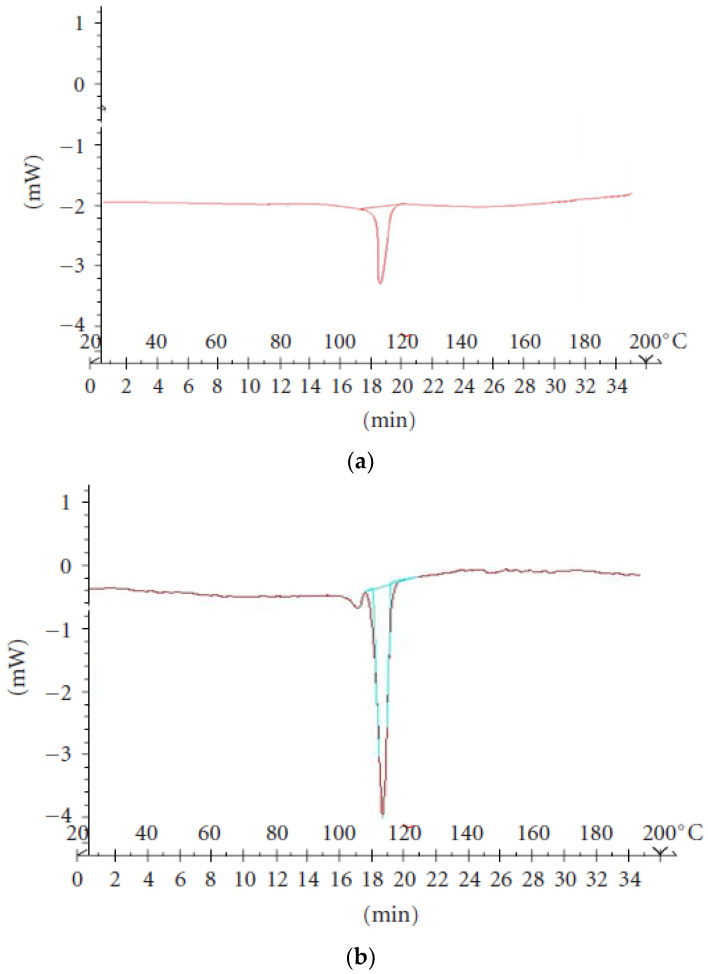
DSC thermogram of (**a**) pure VS and (**b**) physical mixture of VS.

DSC thermograms for pure VS and VS formulation were recorded using a DSC analyzer, and the spectra are shown in Figure 2. Obtained spectra were interrelated to find out the Exo and endothermic peaks. Based on these observations, it was evident that pure VS and VS formulation showed an endothermic peak at 113 °C and 116.5 °C, respectively, and no added peaks were observed. This confirms the compatibility (as there is no change in the crystalline form of VS).

### 2.2. Step I: Optimization of Preparation of Valsartan Nanocrystals

BBD was used to investigate the impact of selected variables and their interactions in resulting in the minimum PS, PDI, and maximum zeta potential. A total of 17 experimental trials were projected, and their observed responses were given in Table 2. The PS of experimental formulations was identified in the range of 526.5 to 243.6 nm. Zeta potential, which will determine formulations’ stability, ranged between 15.52 to 39.25 mV. The experimental results were analyzed for selected responses using f_x_ model and ANOVA.

The quadratic model was selected for both the responses based on the sequential sum of squares (Type-I) and fit summary. Model F-value, *p*-value, and R^2^ values were considered for selecting the model. Additionally, the quadratic model has the highest polynomial order with a *p*-value (level of significance) of <0.0001 (Table 3).

For PS, the predicted R² of 0.9423 is in reasonable agreement with the adjusted R² of 0.9917 (i.e., the difference is less than 0.2). Adequate precision measures the signal-to-noise ratio. A ratio greater than four is desirable. The ratio of 51.052 indicates an adequate signal. This model can be used to navigate the design space. Similar results were observed for PDI and zeta potential (0.9390, 0.9804 and 35.781; 0.9261, 0.9889 and 46.676) [38].

**Figure 3:**
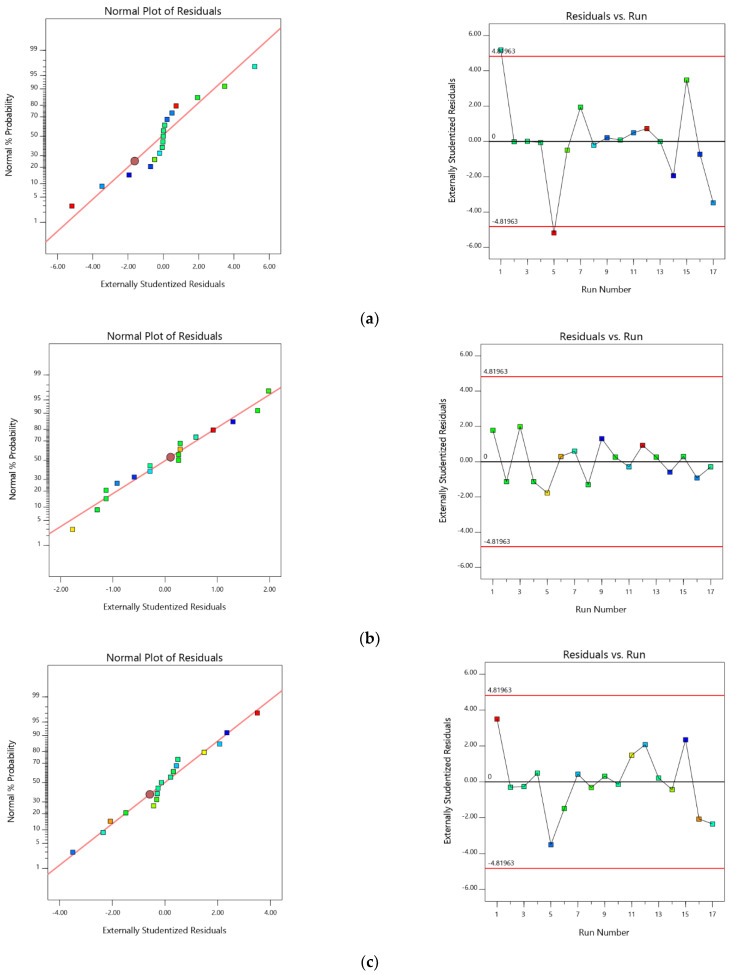
Normal probability and residuals plots for (**a**) PS (**b**) PDI, and (**c**) zeta potential. (Color dots represents Run number).

The accuracy of all of these selected models was further confirmed by the normal plot of residuals [39]. The prescribed statistical application will not be applied for this as the visual inspection graph is acceptable [40,41]. For all of the selected responses, all of the studentized residuals were distributed nearer to the straight line, confirming that the chosen model can be accepted statistically [42,43]. Figure 3 signifies the experimental run against the residuals as a process of recognizing the lurking variables that affect the responses. A scattered trend was detected within the prescribed limit, thus representing the time-coupled variable slink in the background. The reproducibility of the experiments ensures accurate results and guarantees transparency in understanding the methodology, which can be confirmed with the coefficient of variation (CV) value. As the required CV value was comparatively lower (3.24% for PS; 2.64% for PDI & 2.86% for zeta potential) than prescribed (CV < 10%), the consistency and precision of the design were ensured. An additional parameter, lack of fit, measures the inability of the model to represent the complete data [44]. As evident from the ANOVA data, the lack of fit was found to be non-significant (*p* > 0.05) in confirming the fitness of the selected design.

ANOVA was performed to study the impact of quantitative effects of selected factors on responses. Obtained data were subjected to multiple regression to yield polynomial equations. The Model F-values of 214.68, 90.00, and 158.71 implies that all of the selected models are significant.

In the case of particle size, A, B, C, AB, AC, BC, A², B², and C² are significant model terms. The experimental design indicated that PS was potentially affected by (i) antagonist effect of factor A, C, AB, BC, polynomial terms of B and C with a *p*-value of <0.0001, <0.0001, <0.0001, 0.0039, and <0.0001, respectively; and (ii) synergistic effect of B, AC, and polynomial terms of A (the effects of A being the highest). The experimental design indicated that PDI was potentially affected by (i) antagonist effect of factor A, B, C, AB, polynomial terms of B and C [polynomial term]; and (ii) synergistic effect of AC, BC, and polynomial terms of A, (the effects of A being the highest). ANOVA results confirm that the Zeta potential will highly depend on the (i) antagonist effect of factor B, polynomial terms of C; and (ii) synergistic effect of A, C, AC, and polynomial terms of A, B (the effects of A being the highest). All of the factors have shown the *p*-value < 0.0001 except for the polynomial terms of C (0.0013) (Table 4).

Equations generated for coded factors,

PS = +351.34 − 87.46 A + 19.61 B − 39.85 C − 40.30 AB + 24.33 AC − 8.62 BC + 51.56 A² − 15.04 B² − 35.87 C²

PDI = +0.3180 − 0.0500 A − 0.0138 B − 0.0413 C − 0.0125 AB + 0.0175 AC + 0.0050 BC + 0.0410 A² − 0.0215 B² − 0.0415 C²

Zeta Potential = +23.93 + 6.21 A − 3.59 B + 2.09 C + 0.6450 AB + 4.47 AC + 0.7425 BC + 1.25 A² + 4.20 B² − 1.67 C²

**Figure 4:**
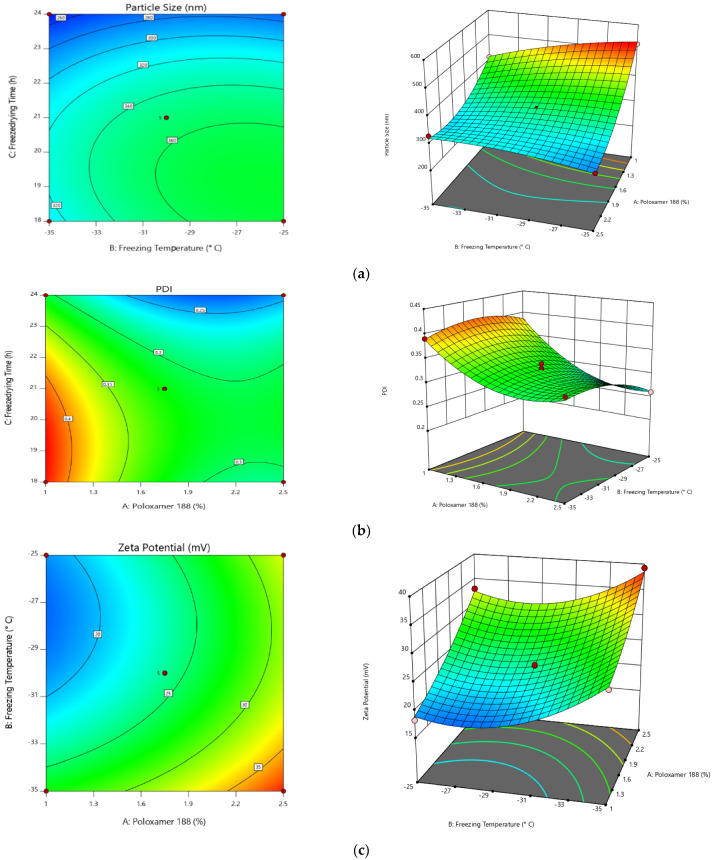
RSG for (**a**) PS, (**b**) PDI, and (**c**) zeta potential.

All of the above equations can be applied to predict the response for any given concentrations of the selected factors. Factor coefficients additionally help in comparing their relative impact on the responses. Contour plots and 3 D RSG (response surface graphs) are crucial for explaining both the interaction and main effect. The measured responses are pictured with these graphs (Figure 4).

**Figure 5:**
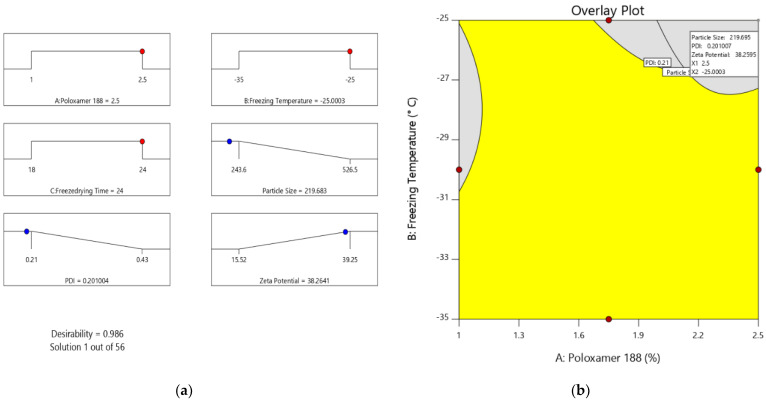
(**a**) Ramp chart and (**b**) overlay of optimization result. (Red color dots represents optimized concentrations of selected variables and blue dots represents for optimized result of chosen responses).

Optimization of different series of models obtained from the experimental analysis can be carried out by applying the desirability function [D]. To plot the overlay graph, each response was set to various limits, particle sizes, and zeta potential- maximums and PDI-minimums [45,46]. All of the selected variables were involved in the design space. The combined desirability plot for all of the responses has shown a maximum D value of 0.986, which was obtained at optimum concentrations of independent variables (Figure 5a), and the critical responses were overlayed in a contour plot (Figure 5b) [47]. Based on this desirability approach, a formulation containing 2.5% of poloxamer, a freezing temperature of −25 °C, and 24 h of freeze-drying time can accomplish the prerequisites of the optimized formulation. Furthermore, NC formation can be confirmed by surface morphology study using scanning electron microscopy (Supplementary Figure S1).

Consequently, these optimized concentrations can result in a PS of 219.68 nm, 0.201 PDI-, and zeta potential of −38.26 mV. By using these concentrations, optimized formulation (O-VNC) was prepared and evaluated. The experimental results were compared with theoretical values to validate the experiential design. The relative error was less than 2%, which confirms the preciseness of the design and high degree of internal consistency [48].

Step-II and III: Preparation and coating of VNM.

O-VNC were further compressed and coated to form VNM and further evaluated for weight variation, hardness, friability, and disintegration (Table 5).

Step IV: Preparation of fast dissolving tablets of valsartan (F-VS).

Prepared F-VS were evaluated for weight variation, hardness, friability, and disintegration. The results are depicted in Table 6. Based on the disintegration time, F-VS3 was selected to fabricate the final chronotherapeutic drug delivery system.

#### 2.2.1. Dissolution Study

**Figure 6:**
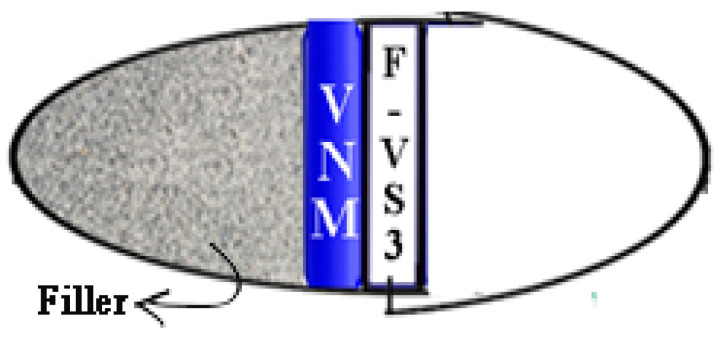
“Tab in cap” system for VS with filler (Lactose-200 mg).

**Figure 7:**
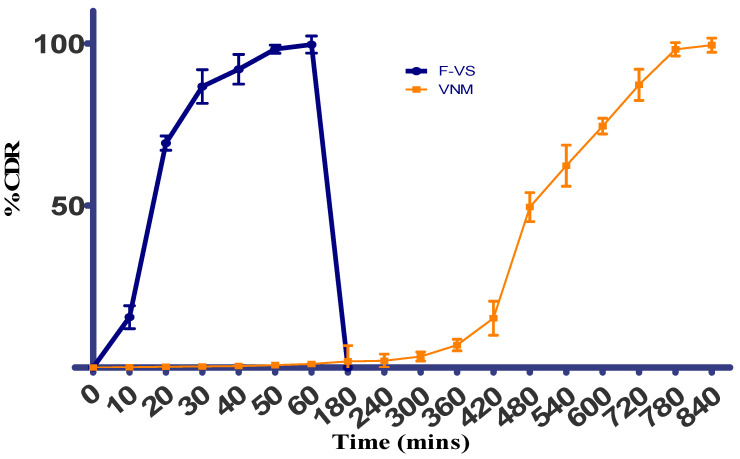
Bipulse drug release of tablet in capsule system.

The final chronotherapeutic drug delivery system was designed using VNM and F-VS3 as described in Section 3.10 and studied for its dissolution profile. As shown in Figure 6, a pulse drug release was identified [49]. Initially, the uncoated capsule cap will dissolve, followed by VS from F-VS3 to induce a first pulse rug release. In 60 min, complete VS release was observed. During this time, there was a slight VS release from the VNM, owing to the impermeability of the capsule body. As required, the second pulse of VS release was observed after a lag time of 5 h, and 98.26% of drug release was observed at the end of the study (Figure 7). A formulation developed by Nayak et al., showed 5–6 h lag time and 10 ± 2.1% drug release in the initial 6 h following rapid release (99 ± 1.7% release in 12 h) [25]. It has been claimed that swelling and relaxation of ethylcellulose films will increase with the HPMC concentration. HPMC usually hydrates and thus produces some pores within the film. Additionally, some HPMC will migrate to the dissolution medium and create a few regions to enhance the permeability of the drug, which finally enhances the dissolution profile.

#### 2.2.2. Stability Studies

No change in the physical appearance of the capsule was observed in both the storage conditions throughout the study period. The dissolution profile of the test samples was compared by calculating similarity and dissimilarity factors using the optimized formulation as a reference to ensure the same bi-pulse release with a specified lag time. As required, all of the samples showed an excellent similarity profile (>90) concerning the reference formulation (Table 7).

Conclusively, this demonstrated the use of ICH Q8 (R2) pharmaceutical development guidelines for robust product development using the principles of quality by design.

## 3. Materials and Methods

### 3.1. Materials

VS with 99.7% assay [Mol. Weight-Mw 435.5297 g/mol] was obtained as gift samples from Ranbaxy Laboratories Ltd., Gurgaon, Haryana, India. BASF (USA) gifted HPMC E5 (Mw-1261.4297 g/mol), ethylcellulose (Mw-454.5/mol). SSG (Sodium starch glycolate) (Mw-747.76), Polyplasdone XL (Mw-177.12297 g/mol), Microcrystalline cellulose (MCC) (Mw-342.297 g/mol), talc, magnesium stearate, and HPMC K15 M were purchased from Loba Chemie Pvt Ltd., Mumbai, India. The purity of all of the reagents was maintained between 99.2–99.7%. All other chemicals and solvents used were of analytical grade, procured from Merck Ltd., Mumbai, India.

### 3.2. Formulation Development of “Tab in Cap” System for VS

“Tab in cap” can be fabricated in four various stages: (1) optimization of preparation of VS nanocrystals [VS-NC]; (2) preparation and coating of nanocrystal compressed tablets [VNM]; (3) preparation of Fast dissolving tablets of VS [F-VS]; and (4) filling/assembly of mini-tablets of VNM and F-VS into coated size 1 capsule (Average fill weight 400–500 mg. lock length—19.30 mm, External diameter: Cap—6.91 mm; Body—6.63 mm). A schematic diagram of the “tab in cap” system of VS to achieve pulse drug release is shown in Figure 6.

### 3.3. Compatibility Studies 

#### 3.3.1. Fourier Transform Infrared Spectroscopy (FTIR)

Pure VS and VS with all excipients were subjected to FTIR spectroscopic studies (Shimadzu IR Affinity-1, Digital IR Spectrometer, Japan) using the KBr pellet method in a range between 4000 and 450 cm^−1^ to identify the interactivity [49,50].

#### 3.3.2. Differential Scanning Calorimetry (DSC) Studies

DSC study was conducted for both pure VS and VS with all excipients. DSC scans were obtained by utilizing an organized thermal analyzer system (DSC 60 Shimadzu Corporation, Tokyo, Japan). Indium was used as a reference to carry out temperature standardization. For all of the samples, sealed and punctured aluminum pans were utilized in the study. The entire samples were set up at a scanning rate of 10 °C per minute from 20–200 °C.

### 3.4. Step I: Optimization of Preparation of Valsartan Nanocrystals

VS-NC were fabricated using modified anti-solvent precipitation. The defined quantity of VS was mixed and dissolved in 20 mL of ethyl alcohol at ambient temperature. Different concentrations (1–2.5%) of Poloxamer-188 were used to prepare 30 mL of solution with purified water and then rested in the ultrasonicator to sustain the temperature of the solution in the range of 5–10 °C. A mechanical stirrer was placed into the solution, and the stirrer rate was put down to 1000 rotations/min for 10 min and subsequently by 500 rpm for 1 h to obtain a uniform solution. The prepared VS solution was added to the pre-cooled Poloxamer-188 solution through the 18-gauge needle to form nanosuspension initially. The formed nanosuspension was filtered using Whatman filter paper and cleaned using water. The filtrate was frozen and then dried at various conditions to obtain a free-flowing powder.

#### Optimization

Preparation of VS-NC is optimized through the statistical method RSM. The concentration of poloxamer 188 (X_1_), freezing temperature (*X*_2_), and freeze-drying time (*X*_3_) were selected as independent variables at three different levels are coded as -1 (low), 0 (medium), and +1 (high). All of these variables were studied for their interaction for particle size (PS), polydispersity index (PDI), and zeta potential using BBD of Design Expert 12 (Stat Ease Inc., Minneapolis, USA), producing 17 experimental trails. Table 8 exhibits the entire plan of the experiment, the interns of coded and actual values of the chosen variables, and the limitations of selected responses. Further, polynomial equations generated were validated using ANOVA (Analysis of variance). Additionally, several statistical tools were applied to all of the experimental runs in selecting the best fit model. In each trial, a quadratic design was employed to compute the response, and further regression analysis was carried out.

$$Y_{i\left(Quadratic\right)}=b_{0}+b_{1}X_{1}+b_{2}X_{2}+b_{3}X_{3}+b_{4}X_{1}X_{2}+b_{5}X_{1}X_{3}+b_{6}X_{2}X_{3}+b_{7}X_{1}^{2}+b_{8}X_{2}^{2}+b_{9}X_{3}^{2}$$

where, *Y_i_*—Selected response or dependent variable, *b_o_*—Arithmetic response, *b_i_*—The estimated coefficient for main effects (*X*_1_, *X*_2_, *X*_3_); interaction terms of main effects (*X*_1_*X*_2_, *X*_2_*X*_3_, *X*_1_*X*_3_), and polynomial terms of independent variables (*X*_1_^2^, *X*_2_^2^, *X*_3_^2^).

### 3.5. Characterization

PS, PDI, and zeta potential.

The VS-NC’s average PS, PDI, and electrokinetic potential were examined through the dynamic light scattering technique (DLS) by Malvern Zetasizer (2000, UK). The specified quantity of VS-NC was re-dispersed into an abundant measure of milli-Q water, and vortex stirred for 5 min to avoid the occlusion of particles [51]. The end sample was examined for 1 min in triplicate at 25 °C.

### 3.6. Step II: Preparation of Valsartan Nanocrystal Minitablets

VS-NC was further compressed to mini-tablets, using nine station punching machine (Rimek Mini Press, Karnavati Engineering, Gujarat, India). As shown in the formulation table, VS-NC were blended uniformly with the excipients (Table 6). Hardness was maintained in the range of 6–8 kg/cm^2^.

### 3.7. Step III: Coating of Core Mini Tablets 

The coating solution 4% *w/v* was made by dissolving ethyl cellulose and HPMC E 50 in 3:1 proportion with isopropanol. In vitro, standardized minitablets were subjected to coating in Labs coat M1 (Shakti Pharmatech Pvt Ltd., Gujarat, India) [52]. The process parameters maintained were as follows:

Inlet and product temperature: 55 °C and 30–35 °C

Pan size and speed: 12 Inch and 30 rpm

Batch size: 500 gm

Spraying rate and atomizing pressure: 8 g/min and 38 psi.

### 3.8. Step IV: Preparation of Fast Dissolving Tablets of Valsartan

Fast dissolving tablets of VS are fabricated to give immediate pharmacological effect. The composition of F-VS is shown in Table 9. The prepared blend was examined for pre-compression parameters, and later, directly compressed to retain the hardness within the 3–4 kg/cm^2^ range.

### 3.9. Evaluation Tests for Prepared Tablets

The compressed tablet dosage forms were examined for physical characteristics such as hardness, thickness, weight variation, and friability. Hardness and friability were checked by Monsanto hardness tester and Roche friabilator (Electrolab-EF-2). Correspondingly, thickness and weight variation were considered with digital screw gauge and electronic balance (Citizen scales Pvt. Ltd., India).

#### Disintegration Test

A disintegration test for F-VS was conducted using 900 mL of pure water as a medium and is maintained at 37 ± 0.5 °C. Six randomly picked tablets were kept in the glass tubes and operated. The mean time needed for the complete disintegration of tablets must be recorded as disintegration time (*n* = 6). There should be no residue remaining on the sieve (#10).

### 3.10. Filling/Assembly of Mini-Tablets and F-VS into Size 1 Capsule

Hard gelatin capsules of size 1 were selected, and then caps and bodies were separated. Coating solution of prepared by using 10% *w/v* ethyl cellulose in methanol and 0.5% 0.5% dibutyl phthalate (plasticizer) was added [53]. Only bodies were allowed to dip in the prepared coating solution. The coating process was optimized (double coating with 60 min drying time) to maintain the capsule integrity to meet the requisites of a chronotherapeutic drug delivery system. The optimized formulation of VNM and selected F-VS were filled into the capsule. At first, the capsule was filled with lactose (200 mg) as filler at the bottom. Also, both VNM and F-VS were placed accordingly. Later the cap is placed properly and sealed with the body. Finally, the tab in cap system was evaluated for in-vitro dissolution studies to confirm the pulsatile drug release.

### 3.11. In Vitro Dissolution Study

The final Tab in Cap system was studied for in vitro drug release using Electro Lab dissolution apparatus (EDT O081X), of USP type I at 50 rpm. In vitro dissolution conditions maintained are of; Volume and temperature of medium: 900 mL and 37 ± 0.5 °C; pH of the medium: 0.1 N HCl (pH 1.2) for 2 h, followed by phosphate buffer pH 4.5 for 3 h, and then shifted to phosphate buffer (pH 6.8) for the remaining term. At defined time intervals, the sample was collected, filtered, and analyzed for absorbance at 248 nm.

### 3.12. Stability Studies

The short duration stability test was conducted for tab in cap system. Adequate numbers of capsules were filled into amber glass bottles and sealed using a rubber cork stopper. The glass bottles are then loaded into stability chambers that were preserved at 40 °C ± 2 °C + 75% RH ± 5% RH and 25 °C ± 2 °C + 60% RH ± 5% RH around 6 months. Test samples were drawn at different time-lapse and were examined for physical characteristics and in-vitro drug release study. The data of the in-vitro dissolution study was collated against the drug release pattern at zero time by similarity factor (*f*_2_) to observe the deviations.

## 4. Conclusions

Valsartan nanocrystals were successfully formulated using the modified anti-solvent precipitation method, using the design of experiments approach to enhance apparent solubility and dissolution. The desirability approach demonstrated that the formulation with 2.5% of poloxamer, freezing temperature of −25° C, and 24 h of freeze-drying time fulfilled the requirements in attaining minimum PS, PDI, and maximum zeta potential. Optimized nanocrystal formulation was compressed into minitablets and then coated with ethyl cellulose and HPMC E 50. The selected fast dissolving tablet formulation was filled with coated nanocrystal minitablet into the capsule of size 1. A bi-pulse drug release profile was observed with a lag time of 5 h. The polymers used for nanocrystals coating are responsible for delaying the release. Thus, this approach can be most promising for pulsatile or chronotherapeutic drug release of valsartan to overcome morning surges.

## Figures and Tables

**Table 1:** Characteristic peaks and the possible group assigned for different samples.

Sample	Frequency cm^−1^	Group Assigned
VS	3447	N-H functional group
	2963	C-H group stretching
	1731	Carboxyl carbonyl
	1603	Amide carbonyl group
	1514	C=C aromatic group
	1273	C-O stretch
VS formulation	3425	N-H functional group
	2962	C-H group stretching
	1730	Carboxyl carbonyl
	1603	Amide carbonyl group
	1513	C=C aromatic group
	1272	C-O stretch

**Table 2:** Experimental runs projected and their responses observed.

Run	A: Poloxamer 188	B: Freezing Temperature	C: Freeze Drying Time	Particle Size	PDI	Zeta Potential
	%	°C	h	nm		mV
6	1	−35	21	413.5	0.39	26.95
5	1	−25	21	526.5	0.38	18.21
12	1	−30	18	521.4	0.43	20.24
15	1	−30	24	398.2	0.31	15.52
8	1.75	−35	18	311.2	0.31	28.59
7	1.75	−25	18	374.5	0.28	20.18
14	1.75	−35	24	243.6	0.22	31.24
9	1.75	−25	24	272.4	0.21	25.8
3	1.75	−30	21	351.4	0.33	23.76
13	1.75	−30	21	351.3	0.32	24.06
2	1.75	−30	21	351.2	0.31	23.74
10	1.75	−30	21	351.9	0.32	23.84
4	1.75	−30	21	350.9	0.31	24.23
1	2.5	−35	21	329.8	0.32	39.25
11	2.5	−25	21	281.6	0.26	33.09
17	2.5	−30	18	287.2	0.29	22.56
16	2.5	−30	24	261.3	0.24	35.71

**Table 3:** Model statistical summary.

Response	Models	R^2^	Adju.R^2^	Pred.R^2^	Adequate Precision	Sequential *p*-Value	Remarks
PS	Linear	0.7459	0.6873	0.4682	----	0.0004	
	2 FI	0.8347	0.7356	0.1509	51.052	0.2125	
	Quadratic	0.9964	0.9917	0.9423	---	<0.0001	Suggested
	Cubic	1.0000	1.0000		---	<0.0001	Aliased
PDI	Linear	0.6614	0.5833	0.3061	---	0.0022	
	2 FI	0.6981	0.5170	−0.4893	---	0.7524	
	Quadratic	0.9914	0.9804	0.9390	35.781	<0.0001	Suggested
	Cubic	0.9947	0.9789		---	0.5413	
	Linear	0.7151	0.6494	0.4283		0.0008	
Zeta potential	2 FI	0.8491	0.7585	0.3272		0.0842	
	Quadratic	0.9951	0.9889	0.9261	46.676	<0.0001	Suggested
	Cubic	0.9997	0.9988			0.0064	

**Table 4:** Analysis of variance (ANOVA) results.

	Intercept	A	B	C	AB	AC	BC	A²	B²	C²
**Particle Size**	351.34	−87.4625	19.6125	−39.85	−40.3	24.325	−8.625	51.555	−15.045	−35.87
***p*-values**		< 0.0001	0.0001	<0.0001	<0.0001	0.0003	0.0500	<0.0001	0.0039	<0.0001
**PDI**	0.318	−0.05	−0.01375	−0.04125	−0.0125	0.0175	0.005	0.041	−0.0215	−0.0415
***p*-values**		< 0.0001	0.0019	<0.0001	0.0173	0.0034	0.2548	<0.0001	0.0009	<0.0001
**Zeta Potential**	23.926	6.21125	−3.59375	2.0875	0.645	4.4675	0.7425	1.252	4.197	−1.6705
***p*-values**		<0.0001	< 0.0001	<0.0001	0.0915	<0.0001	0.0591	0.0059	<0.0001	0.0013

**Table 5:** Post compression parameters for VNM and F-VS.

Formulation	Average Weight (mg)	Weight Variation (%)	Hardness (kg/cm^2^)	Friability (%)	Disintegration (min)
VNM	101	±1.25	7	0.154	368
F-VS1	100	±0.85	4	0.268	14
F-VS2	101	±1.55	5	0.280	9
F-VS3	101	±1.25	4	0.325	8
F-VS4	100	±0.65	4	0.325	16

**Table 6:** Formulation of VNM.

S. No	Formulation Code	VS-NC	MCC (PH 102)	Talc	Magnesium Stearate	HPMC K 15 M
1.	VNM-1	10 mg	64.2 mg	0.4 mg	0.4 mg	25 mg

**Table 7:** Stability studies for tablet in capsule system.

Test	Initial	25 °C ± 2 °C + 60% ± 5% RH		40 °C ± 2 °C + 75% ± 5% RH	
		3 M	6 M	3 M	6 M
Capsule physical appearance	Complies	Complies	Complies	Complies	Complies
*f*_2_	--	96.08	94.25	95.28	93.25

**Table 8:** Experimental plan for BBD in terms of actual and coded values.

Factors/Independent Variables	Levels			Responses/Dependent Variables	Constraints
	−1	0	+1		
Concentration of Poloxamer 188 (%)—*X*_1_	6	8	10	Particle size (nm)	Minimum
Freezing Temperature (°C)—*X*_2_	70	80	90	PDI	Minimum
Freeze drying Time (h)—*X*_3_	1.5	2	2.5	Zeta Potential (mV)	Maximum

**Table 9:** Composition of F-VS.

S. No	Material	Quantity
1.	VS	40
2.	Polyplasdone XL and SSG	2 mg, 6 mg (F-VS1,F-VS2) and 3 mg, 9 mg (F-VS3, F-VS4)
3.	Spray-dried lactose	55 mg (F-VS1), 51 mg (F-VS2) 54 mg (F-VS3) and 48 mg (F-VS4)
4.	Magnesium stearate	1 mg
5.	Talc	2 mg
	Total tablet weight	100 mg

## Data Availability

Data is contained within the article and Supplementary Materials.

## Supplementary Materials

The following supporting information can be downloaded at: https://www.mdpi.com/article/10.3390/ph15030311/s1, Figure S1: Scanning electron microscopy image of optimized nanocrystal formulation.
