# Supplementary figures and images for: Development of Nanocrystal Compressed Minitablets for Chronotherapeutic Drug Delivery
Source: Pharmaceuticals (Basel). 2022 Mar 4;15(3):311. doi: 10.3390/ph15030311 (PMC8950040; doi:10.3390/ph15030311)

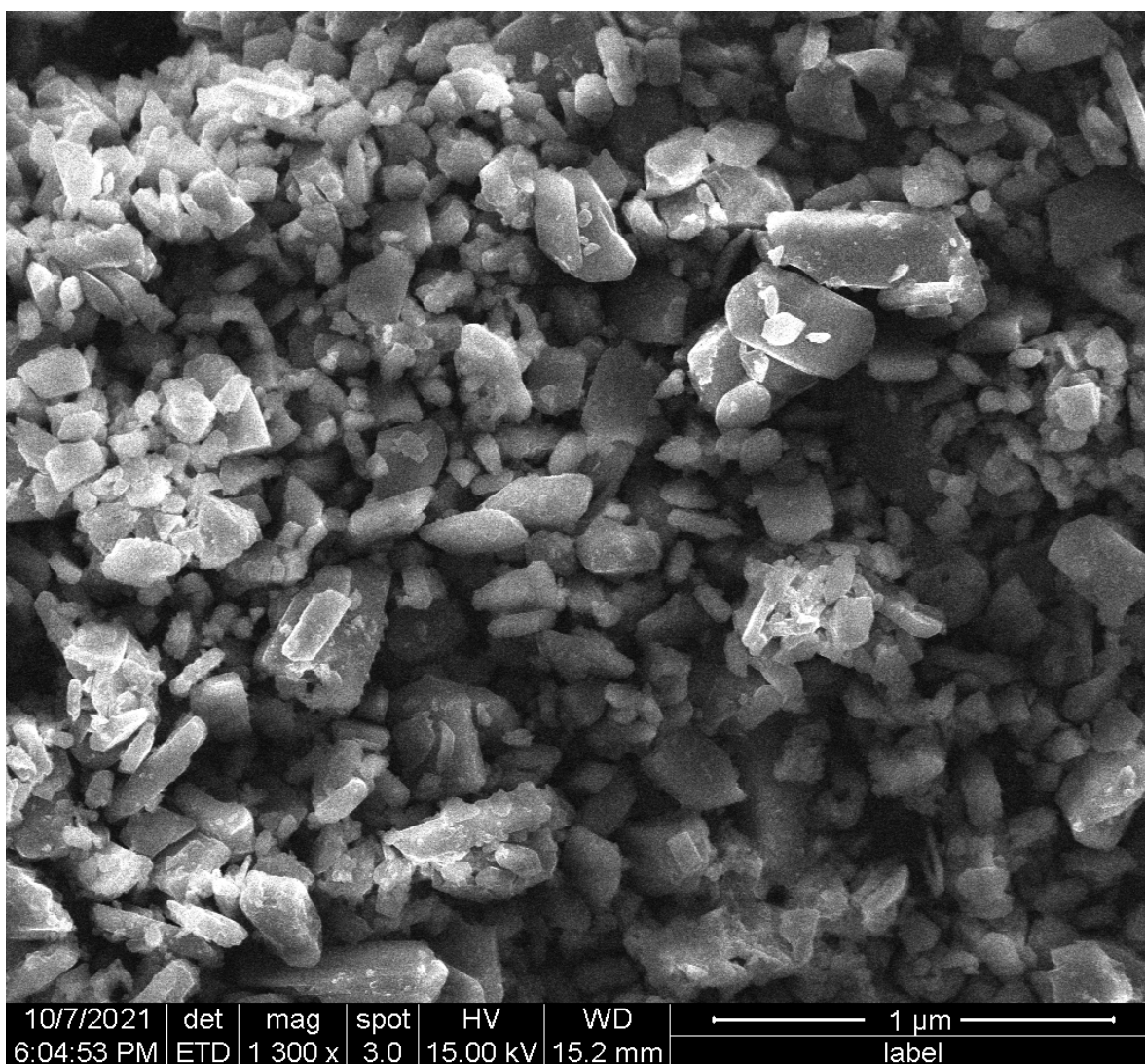

**Figure S1.** Scanning electron microscopy image of optimized nanocrystal formulation.

Supplement: Supplementary file 1 [file pharmaceuticals-15-00311-s001.zip › pharmaceuticals-1590783-supplementary.pdf]
